# Supplementary material for: Identification of Secreted Protein Gene-Based SNP Markers Associated with Virulence Phenotypes of Puccinia striiformis f. sp. tritici, the Wheat Stripe Rust Pathogen
Source: Int J Mol Sci. 2022 Apr 8;23(8):4114. doi: 10.3390/ijms23084114 (PMC9033109; doi:10.3390/ijms23084114)
Supplement: Supplementary file 1 [file ijms-23-04114-s001.zip › SP-SNP SupFigR.pptx]

## Slide 1
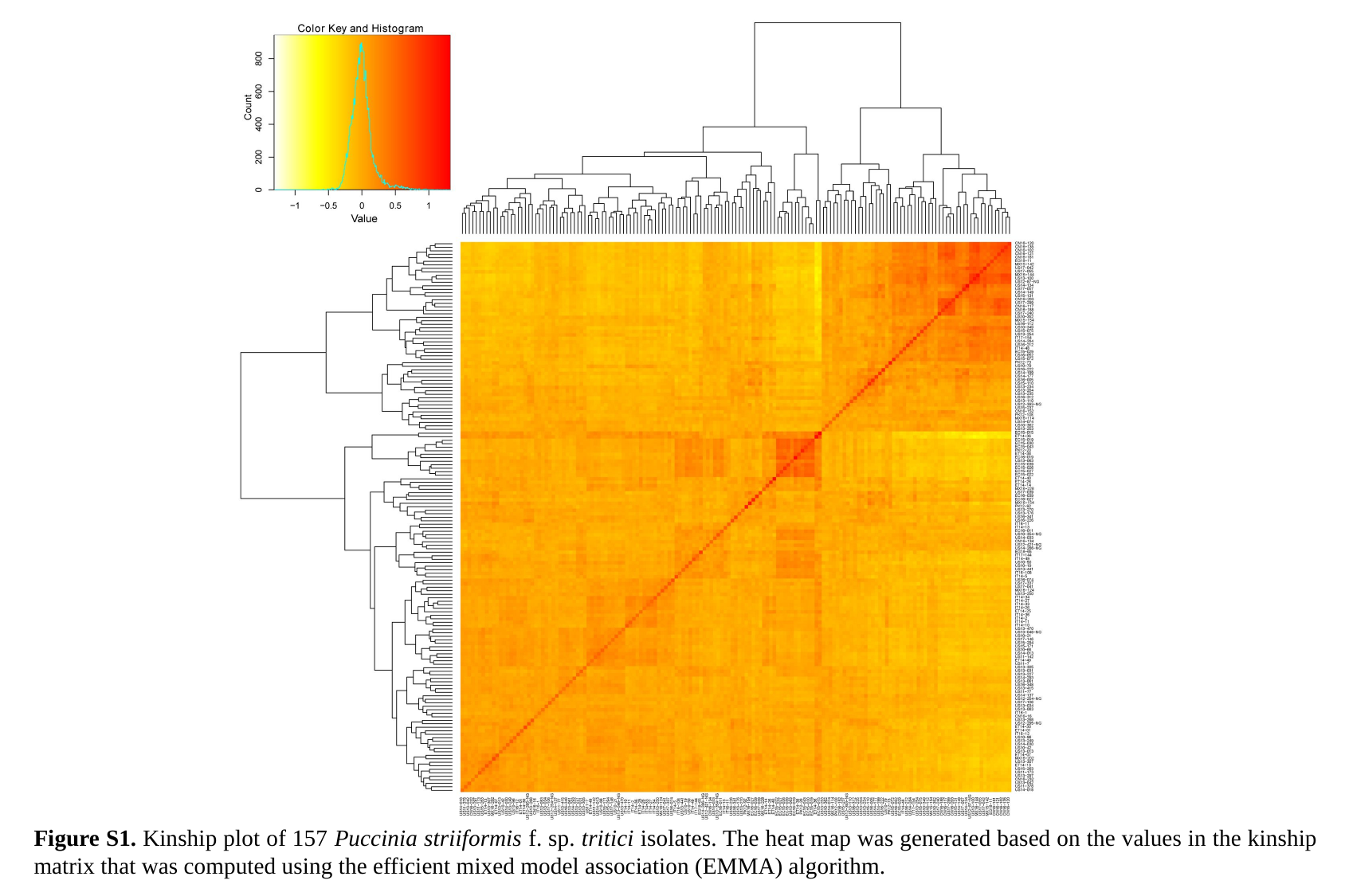

Figure S1. Kinship plot of 157 Puccinia striiformis f. sp. tritici isolates. The heat map was generated based on the values in the kinship matrix that was computed using the efficient mixed model association (EMMA) algorithm.

## Slide 2
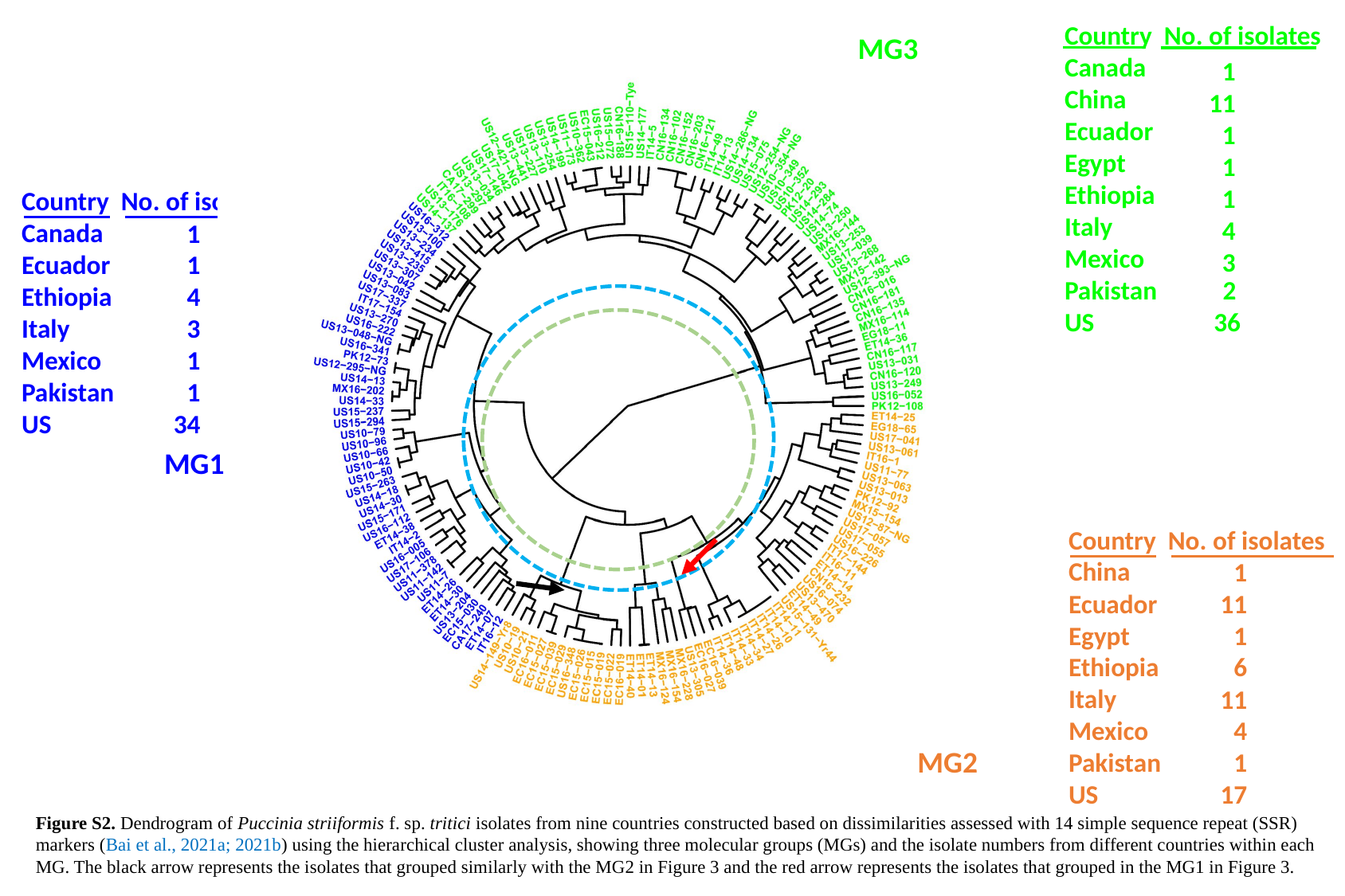

MG3
MG1
MG2
Country No. of isolates
Canada
China
Ecuador
Egypt
Ethiopia
Italy
Mexico
Pakistan 2
US 36
1
11
1
1
1
4
3
Country No. of isolates
Canada
Ecuador
Ethiopia
Italy
Mexico
Pakistan
US
1
1
4
3
1
1
34
Country No. of isolates
China
Ecuador
Egypt
Ethiopia
Italy
Mexico
Pakistan
US
1
11
1
6
11
4
1
17
Figure S2. Dendrogram of Puccinia striiformis f. sp. tritici isolates from nine countries constructed based on dissimilarities assessed with 14 simple sequence repeat (SSR) markers (Bai et al., 2021a; 2021b) using the hierarchical cluster analysis, showing three molecular groups (MGs) and the isolate numbers from different countries within each MG. The black arrow represents the isolates that grouped similarly with the MG2 in Figure 3 and the red arrow represents the isolates that grouped in the MG1 in Figure 3.
